# Utilization of Mechanistic Enzymology to Evaluate the Significance of ADP Binding to Human Lon Protease

**DOI:** 10.3389/fmolb.2017.00047

**Published:** 2017-07-11

**Authors:** Jennifer Fishovitz, Zhou Sha, Sujatha Chilakala, Iteen Cheng, Yan Xu, Irene Lee

**Affiliations:** ^1^Department of Chemistry and Physics, Saint Mary's College Notre Dame, IN, United States; ^2^Department of Chemistry, Case Western Reserve University Cleveland, OH, United States; ^3^Department of Chemistry, Cleveland State University Cleveland, OH, United States

**Keywords:** ADP affinity, Lon protease, ADP-ATP exchange mechanism, steady-state kinetic, nucleotide induced conformational changes

## Abstract

Lon, also known as Protease La, is one of the simplest ATP-dependent proteases. It is a homooligomeric enzyme comprised of an ATPase domain and a proteolytic domain in each enzyme subunit. Despite sharing about 40% sequence identity, human and *Escherichia coli* Lon proteases utilize a highly conserved ATPase domain found in the AAA+ family to catalyze ATP hydrolysis, which is needed to activate protein degradation. In this study, we utilized mechanistic enzymology techniques to show that despite comparable k_cat_ and K_m_ parameters found in the ATPase activity, human and *E. coli* Lon exhibit significantly different susceptibility to ADP inhibition. Due to the low affinity of human Lon for ADP, the conformational changes in human Lon generated from the ATPase cycle are also different. The relatively low affinity of human Lon for ADP cannot be accounted for by reversibility in ATP hydrolysis, as a positional isotope exchange experiment demonstrated both *E. coli* Lon and human Lon catalyzed ATP hydrolysis irreversibly. A limited tryptic digestion study however indicated that human and *E. coli* Lon bind to ADP differently. Taken together, the findings reported in this research article suggest that human Lon is not regulated by a substrate-promoted ADP/ATP exchange mechanism as found in the bacterial enzyme homolog. The drastic difference in structural changes associated with ADP interaction with the two protease homologs offer potential for selective inhibitor design and development through targeting the ATPase sites. In addition to revealing unique mechanistic differences that distinguish human vs. bacterial Lon, this article underscores the benefit of mechanistic enzymology in deciphering the physiological mechanism of action of Lon proteases and perhaps other closely related ATP-dependent proteases in the future.

## Introduction

Lon (protease La) is an ATP-dependent serine protease that is found ubiquitously in nature. In eukaryotes, Lon is localized in the mitochondria and helps maintain proper cellular function, while in prokaryotes it is found in the cytosol (Charette et al., 1981; Chung and Goldberg, 1981; Amerik et al., 1991; Wang et al., 1993, 1994; Goldberg et al., 1994; Suzuki et al., 1995). Lon, like other ATP-dependent proteases such as FtsH, ClpAP, ClpXP, and HslUV, belongs to the AAA+ (ATPase Associated with various cellular Activities) family of proteins. These proteins contain an ATPase domain, which is highly conserved and contains Walker A and B motifs where ATP binding and hydrolysis takes place (Neuwald et al., 1999; Ogura and Wilkinson, 2001). Lon is considered to be one of the simplest proteases because it contains both the ATPase and protease domain in a single subunit (Gottesman and Maurizi, 1992; Maurizi, 1992; Rep and Grivell, 1996).

Lon protease has three activities: intrinsic ATPase, substrate-stimulated ATPase, and ATP-dependent proteolysis. In bacteria, such as *Escherichia coli* (ELon), the main function of Lon is to degrade damaged, irregular and short-lived regulatory proteins in cells in order to maintain proper cellular function (Gottesman and Zipser, 1978; Gottesman et al., 1981; Goldberg and Waxman, 1985; Gottesman and Maurizi, 1992; Maurizi, 1992; Goldberg et al., 1994; Gottesman, 1996). In humans, Lon is critical for maintaining the structure and integrity of mitochondria (Bota et al., 2005). Human Lon (hLon) has been found to selectively degrade accumulating proteins damaged by oxidative stress over their native counterparts (Bota and Davies, 2001, 2002).

Lon preferentially degrades damaged or misfolded proteins at its proteolytic site while the ATP is bound and hydrolyzed into ADP and inorganic phosphate (Pi) at its ATPase site. In ELon, ADP was found to act as an inhibitor that binds to the enzyme with much higher affinity than ATP (Thomas-Wohlever and Lee, 2002). Kinetic studies indicated that ADP release is the rate-limiting step along the reaction pathway of ELon (Menon and Goldberg, 1987a,b; Vineyard et al., 2005). These kinetic studies support the model of ADP/ATP exchange, which shows the enzyme becomes proteolytically “inactive” when ADP is bound (Waxman and Goldberg, 1986; Goldberg et al., 1994). When the protein substrate interacts with Lon at the proteolytic active site, it promotes the release of ADP at the ATPase site, which is considered as the rate-limiting step. Lon is only proteolytically “active” when bound ADP is exchanged with ATP (Menon and Goldberg, 1987b). In bacterial Lon, *in vitro* nucleotide binding and ADP inhibition kinetic studies suggest that the proteolytic activity could be regulated by cellular ATP/ADP level.

Sequence alignment of hLon, ELon, and *Salmonella Typhimurium* Lon revealed that bacterial Lon such as ELon and *S. Typhimurium* Lon share greater than 99% sequence identity. However, they only share 42% identity with hLon, but a much higher sequence homology is found within the ATPase domain (Goldberg et al., 1994; Johnson et al., 2008). Since bacterial and human Lon exhibit high sequence homology in their ATPase sites and comparable steady-state kinetic parameters in ATPase activity (Frase et al., 2006), it is plausible that the substrate-promoted ADP/ATP exchange mechanism found in ELon is also used to regulate the proteolytic activity of human Lon in the mitochondria. As mitochondrial Lon functions to degrade oxidized proteins, it is suggested that the protein substrate will bind Lon allosterically in order to reverse ADP inhibition in mitochondria by promoting ADP release. If this is the case, then the levels of oxidized protein vs. ADP serves to regulate Lon's activity (Bulteau et al., 2005). As such, the ratio of ADP/oxidative proteins in the mitochondria is kept at a constant ratio by Lon degradation in order to maintain balance.

To evaluate the effect of ADP on human Lon peptidase activity, the fluorogenic peptidase assay previously (Lee and Berdis, 2001; Thomas-Wohlever and Lee, 2002) used to perform mechanistic characterization of bacterial Lon was used in this study to determine the inhibition profile of ADP for human Lon. Using a limited tryptic digestion assay (Patterson et al., 2004), the effect of ADP on the structural changes in human Lon was assessed. A positional isotope exchange experiment that was used to determine the reversibility of ATP hydrolysis in ELon was also used to study human Lon.

## Materials and methods

### Materials

Fmoc-protected amino acids, Boc-2-Abz-OH, Fmoc-Lys(Aloc)-Wang resin, Fmoc-Abu-Wang resin, and HBTU were purchased from Advanced ChemTech and NovaBioChem. Tris, IPTG, chromatography media, DTT, Mg(OAc)_2_, trypsin, kanamycin, chloramphenicol, ATP, DMSO, Tween 20, and all other materials were purchased from Fisher, Sigma, and Amresco.

### General methods

All reactions conditions are listed as final concentrations. Enzyme concentrations are reported as monomer concentration as quantified by Bradford Assay (Bradford, 1976) or absorbance at 280 nm using the molar extinction coefficient (Gill and von Hippel, 1989). Synthesis of FRETN 89–98 (fluorescent and non-fluorescent analogs) were performed as previously described (Thomas-Wohlever and Lee, 2002; Frase and Lee, 2007). Peptides were quantified by extinction coefficient at A_280_. All reactions were run at 37°C unless otherwise stated.

### Expression and purification of human Lon protease

Human Lon was expressed and purified as previously described (Frase et al., 2006). with the following modifications. Human Lon expressed in Rosetta (DE3) cells were grown at 37°C in superbroth (SB) containing 30 μg/mL kanamycin and 34 μg/mL chloramphenicol until they reached an OD_600_ of 1.0 at which they were induced with 1 mM IPTG for 1 hr at 37°C. After induction, cells were harvested at 3000 × g at 4°C. Pelleted cells were combined and resuspended in 50 mM KP_i_ lysis buffer (all buffers contain 5 mM BME, 20% glycerol, and 0.01% Tween 20 unless otherwise stated) and lysed in a Dounce homogenizer on ice three times. For complete lysis, cells were sonicated for 5 min in 15 s pulses at 100 V. Cell lysate was cleared by centrifugation at 20,000 × g for 2 h at 4°C. Cleared lysate was immediately loaded onto a P11 cation exchange column (Whatman) equilibrated in lysis buffer and the flow through was collected. The column was then washed with 0.1 M KP_i_ wash buffer until protein was no longer coming off the column. Finally, Lon was eluted with a linear gradient of 0.1 M KP_i_ to 0.5 M KP_i_ buffer, collected in 20 mL fractions. Fractions were tested for protein content with Bradford dye and positive fractions were analyzed by SDS-PAGE. Fractions containing Lon were combined and diluted to a final KP_i_ concentration of 110 mM then loaded onto a DE52 anion exchange column (Whatman) equilibrated in 110 mM KP_i_ buffer. Flow-through of the load was collected and the protein was eluted with 120 mM KP_i_ buffer. Load and elution fractions were analyzed by SDS-PAGE and Lon-positive fractions were combined and concentrated to ~6 mL using Amicon YM-30 MWCO membrane. Protein was loaded onto a Sepharose S-300 gel filtration column equilibrated in hLon storage buffer (50 mM HEPES, 75 mM KP_i_ pH 7, 5 mM DTT, 1 mM Mg(OAc)_2_, 150 mM NaCl, 20% glycerol, 0.01% Tween 20) and eluted with the same buffer. Fractions were analyzed by SDS-PAGE and Lon-positive fractions were combined, concentrated, quantified, aliquoted, and stored at −80°C.

### ADP inhibition of human Lon peptidase activity

Reactions containing 50 mM HEPES (pH 8.0), 5 mM Mg(OAc)_2_, 2 mM DTT, 300 nM hLon and varying amounts of FRETN 89–98 and ADP were initiated by the addition of 50 μM ATP. Peptide cleavage was monitored at 420 nm (λ_ex_ = 320 nm) on a FluoroMax-3 or FluoroMax-4 fluorometer (Horiba Group) at 37°C. The rate of peptide cleavage was determined by the slope of a line tangent to the linear phase of the time course and normalized by the rate of complete peptide cleavage by trypsin. Observed rate constants (*k*_*obs*_) were determined by dividing by the concentration of enzyme. Kinetic parameters were determined by global fitting of the data using the program GraphPad Prism 6 for non-competitive inhibition (Equation 1; Cleland, 1979).

(1) $$\begin{array}{r} k_{obs}=\frac{k_{cat}\times S^{n}}{K^{\prime }\left[1+\frac{I}{K_{is}}\right]+S^{n}\left[1+\frac{I}{K_{ii}}\right]} \end{array}$$

Where *k*_*obs*_ is the observed rate constant for peptide cleavage, *k*_*cat*_ is the maximum rate constant, *S* is peptide substrate concentration, *n* is the Hill coefficient, *K*′ is the observed Michaelis constant for the peptide substrate, *I* is the inhibitor concentration, and *K*_*is*_ and *K*_*ii*_ are the inhibition constants at low and high concentrations of peptide substrate, respectively. *K*′ is converted to the true Michaelis constant (*K*_*m*_) using (Equation 2; Cleland, 1979).

(2) $$\begin{array}{r} \log K_{m}=\frac{\log K^{\prime }}{n} \end{array}$$

### Effect of phosphate on steady-state ATPase activity

Reactions containing 50 mM HEPES (pH 8), 5 mM Mg(OAc)_2_, 2 mM DTT, 150 nM hLon in the absence and presence of 1 mM sodium phosphate (NaP_i_, pH 7.2) were initiated with 1 mM [α-^32^P]ATP and incubated at 37°C. Aliquots were quenched at various time points (0–15 min) in 0.5 N formic acid and 3 μL was spotted on a PEI-cellulose TLC plate and developed in 0.3 M KP_i_ (pH 3.4). The amount of ADP produced was determined from using Equation (3)

(3) $$\begin{array}{r} \left[ADP\right]=\frac{ADP_{DLU}}{\left(ATP_{DLU}+ADP_{DLU}\right)}*{\left[ATP\right]}_{i} \end{array}$$

Where [*ADP*] is amount of ADP produced, *DLU* is density light units quantified and [*ATP*]_*i*_ is the initial concentration of ATP.

### Positional isotope exchange

Isotopically enriched ${\text{H}}_{2}^{18}$O was acquired from Sigma. The ATPase reaction was carried out in 150 μL total volume with 50 mM Tris pH 7.5, 2 mM DTT, 2 mM Mg(OAc)_2_, 25% ${\text{H}}_{2}^{18}$O, 2 mM ATP, 1 μM WT hLon, with and without 8 μM λN, a protein substrate that stimulates the ATPase activity of Lon. A control experiment was conducted in the absence of enzyme. The reaction mixture was incubated at 37°C and quenched with 2 μL 0.5 M EDTA after 120 min. The aqueous layer containing the phosphate was retained by extraction first with phenol-chloroform, then with chloroform alone. Inorganic phosphate (Pi) was purified from the aqueous layer using a 2 cm AG1-X1 ion exchange column in a Pasteur pipet (Hackney et al., 1980). The ion exchange resin was activated by washing first with 4.5 mL of 1 M HCl, and then with H_2_O until the pH was above 4. The same sample was added and the column was washed with an additional 4.5 mL of H_2_O, then with 0.5 mL aliquots of 10 mM HCl until the pH was less than 2.5. The column was eluted with 2.5 mL of 30 mM HCl in 0.5 mL aliquots, which were combined and lyophilized to dryness. Trimethylsilyl phosphate (TMSP) was generating by derivatizing the inorganic phosphate with 10 μL trimethylsilyldiethylamide (TMSDEA) and 100 μL methylene chloride. The isotopic distribution was determined with a Varian gas chromatograph interfaced with a Varian Saturn 2100T lon trap mass spectrometer. A 30 m VF5-MS column was used for separation. The temperature profile began at 60°C, then increased by 20°C/min to 110°C, then 40°C/min to 240°C and held at 240°C for 5 min. The most abundant ion, M-CH_3_ (MW = 300) was monitored. The ion detected after electron impact was (M-CH_3_)^+^ (MW = 299). The experimental relative abundance is calculated using Equation (4)

(4) $$\begin{array}{r} \text{relative }\%\text{ isotope}=\frac{signal_{isotope}}{signal_{primary}} \end{array}$$

The derivatization reagent TMSP has a high natural abundance of ^29^Si and ^30^Si, which can obscure the interpretation of the ^18^O incorporation results (Hackney et al., 1980). This is known as isotopic spillover and can be calculated according to Table 1. To correctly account for the enrichment due to ^18^O, the expected isotopic abundance was subtracted from the experimental abundance. There should be no enhancement at M+1. Any enhancement at M+2 is a result of ^18^O incorporated into the phosphate. The spillover from the species must be subtracted from the higher molecular weight isotopes, in addition to the expected natural abundance.

**Table 1:** Calculations of the percent isotopic enrichment using the natural isotopic abundance of tris(trimethylsilyl)phosphate minus one methyl group.

	**Calculation**	**Result = Enrichment**
M	100–100	0
M+1	Experimental–24.63	M+1^*^
M+2	Experimental–12.64	M+2^*^
M+3	Experimental–2.12–(M+2^*^ × 0.2463)	M+3^*^
M+4	Experimental–0.5–(M+2^*^ × 0.1265)	M+4^*^

* *Indicates enrichment value generated from calculation in same row*.

### Tryptic digests

Trypsin digestion reactions in a mixture containing 6 μM WT hLon or 1.5 μM WT ELon, 50 mM HEPES (pH 8.0), 5 mM Mg(OAc)_2_, 2 mM DTT, 1 mM ADP were initiated by the addition of 1/50 (w/w) or 1/275 (w/w) TPCK (N-*p*-tosyl-L-phenylalanyl chloromethyl ketone)-treated trypsin with respect to Lon. At 0, 15, and 30 min, a 5 μL reaction aliquot was quenched with 5 μg of soybean trypsin inhibitor (SBTI) followed by boiling at 100 °C for 5 min. The quenched reactions were than resolved by 12.5% SDS-PAGE analysis and visualized with Coomassie Brilliant Blue.

## Results and discussion

### ADP inhibition of peptide cleavage by human Lon as a function of peptide concentration

A fluorescent peptide substrate denoted as FRETN 89-98 was used to monitor the inhibition of hLon activity in a continuous peptidase assay. This 11-mer peptide was derived from the sequence of the λN protein (Maurizi, 1987) that contains an anthranilamide donor at one terminus and a 3-nitrotyrosine quencher at the other terminus with a single cleavage site for Lon and one cleavage site for Lon (Lee and Berdis, 2001). Upon cleavage by Lon protease in the presence of ATP hydrolysis, the peptide separates into two pieces, and shows an increase in fluorescence as the quencher is separated from the fluorophore. Protease activity is measured by monitoring fluorescence emission over time. The fluorescent trace contains a short lag phase, followed by a linear phase, then a leveling out of fluorescence indicating substrate depletion. The slope of the linear phase corresponds to the rate of peptide degradation, which can be converted to observed rate constants for comparative studies.

**Figure 1:**
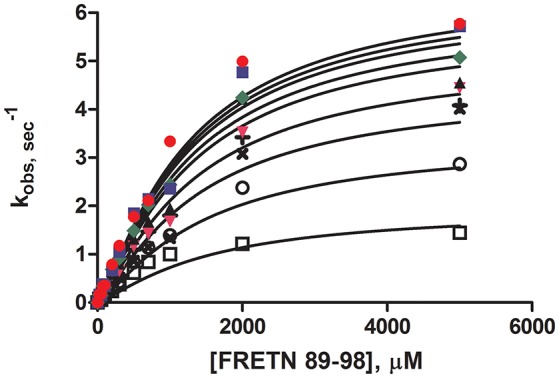
Steady-state ADP inhibition study of WT hLon in the presences of varying FRETN 89–98 concentration. An average of at least three trials for each concentration (0 (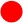), 50 (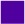), 100 (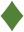), 200 (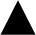), 300 (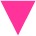), 600 (×), 1000 (+), 2000 (○), 5000 (□) μM of ADP). The plot was fitted with Equation (1), for non-competitive inhibition. The fit yielded the kinetic parameters of K_m_ = 1027 μM, *k*_cat_ = 6.63 sec^−1^, K_ii_ = 1499 μM, K_is_ = 2077 μM, and *n* = 1.25.

Steady-state peptidase time courses were run in the presence of K_m_ level ATP (Frase et al., 2006), varying amounts of peptide substrate and varying amounts of ADP. The rate of each time course was quantified by the slope of a line tangent to the linear phase of the time course. The resulting rate constant data was analyzed using the global fitting programs mentioned in Methods and Materials (Figure 1) to yield the kinetic parameters shown in Table 2. Fitting of the data to Equation (1) or a non-competitive inhibition mechanism yielded inhibition constants of K_is_ ~1500 μM and K_ii_ ~2100 μM. K_is_ and K_ii_ refer to the inhibition constants at low and high concentrations of peptide substrate, respectively. When compared to the parameters determined for bacterial Lon at K_m,ATP_ (K_is_ = 1 μM, K_ii_ = 7 μM; Thomas-Wohlever and Lee, 2002) it can be discerned that ADP binds ~300–1500-fold less tightly to human Lon than it does to bacterial Lon, making its inhibitory effect on peptidase activity less. This result suggests that while ADP release may be rate-limiting in the mechanism of bacterial Lon (Thomas-Wohlever and Lee, 2002; Vineyard et al., 2005), it is more likely human Lon has a different rate-limiting step, a distinction between the mechanisms that must be explored further in the future. The fact that hLon binds to ADP with much reduced affinity than ATP in the presence of a protein substrate such as λN indicates that the substrate-promoted ADP/ATP exchange mechanism found in ELon does not exist in hLon. Additional mechanistic studies directed toward identifying physiological changes in mitochondria that regulates hLon activity are currently underway.

**Table 2:** Kinetic Parameters for ADP inhibition of peptide cleavage by human Lon, determined by curve fitting with the indicated software.

**Parameter**	**Prism 6 (GraphPad)**
*k*_cat_ (sec^−1^)	6.63 ± 0.31
*K*_m_ (μM)	1027 ± 128
*n*	1.25 ± 0.07
*K*_ii_ (μM)	2077 ± 207
*K*_is_ (μM)	1499 ± 381

### Effect of phosphate on steady-state ATPase activity

**Figure 2:**
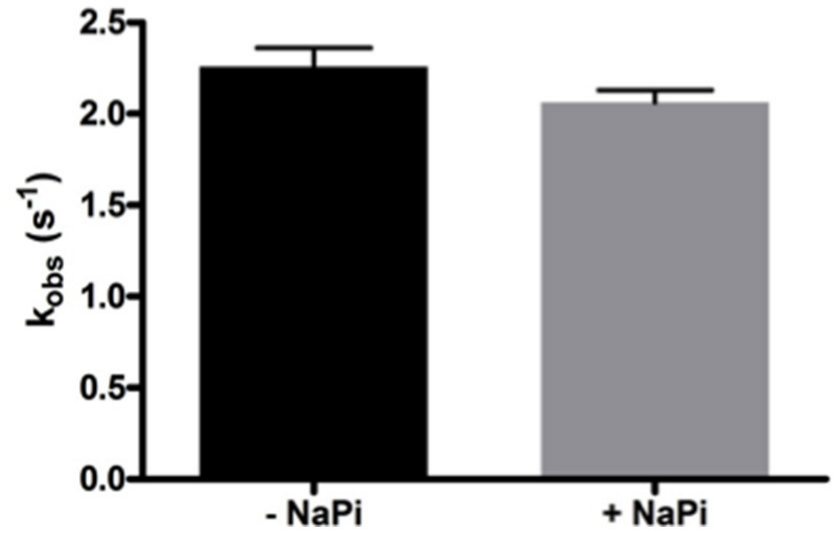
Phosphate does not inhibit the ATPase activity of hLon. Rates of ATP hydrolysis were determined in the absence and presence of 1 mM sodium phosphate. As the rate in the presence of phosphate is not significantly decreased, this confirms that product release is most likely not the rate-limiting step of the mechanism of Lon activity.

As Lon catalyzes the hydrolysis of ATP to yield ADP and inorganic phosphate, phosphate rather than ADP release may limit hLon turnover. The rates of ATPase activity of hLon were measured in the absence and presence of 1 mM sodium phosphate (NaPi) as described in Materials and Methods. As shown in Figure 2, the rate of ATP hydrolysis in the presence of 1 mM NaPi is not significantly inhibited, suggesting the phosphate release is not the rate-limiting step in the mechanism. Combined with the fact that ADP binds very weakly to human Lon, these results indicate that the rate-limiting step is not associated with either of the product release.

### Positional isotope exchange (Scheme 1)

**Scheme 1:**
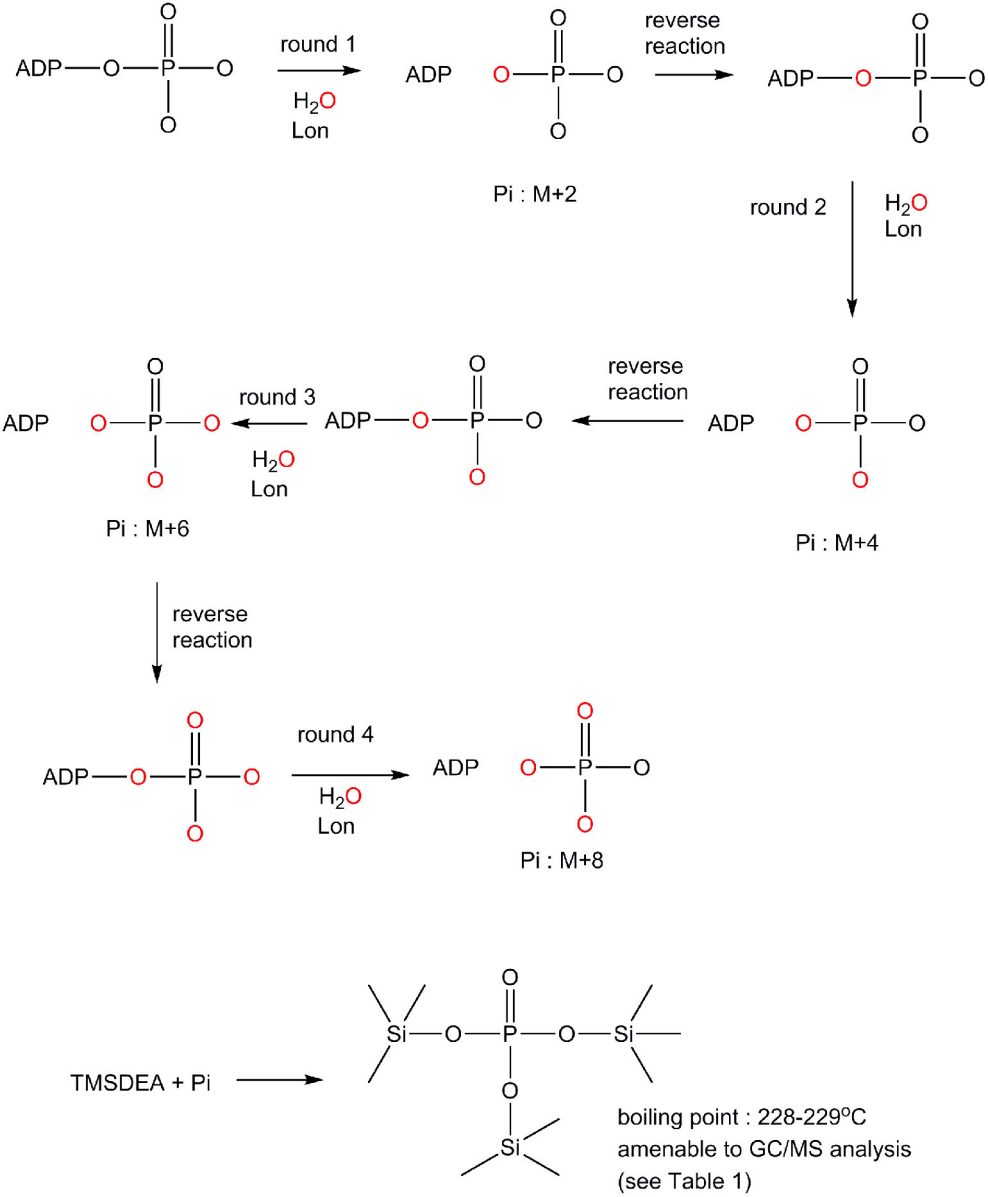
Mechanism by which ^18^O incorporation from ${\text{H}}_{2}^{18}$O into the inorganic phosphate product generated from an ATPase reaction to distinguish an irreversible vs. a reversible ATP hydrolysis mechanism (see text for detail).

The difference in the ADP binding between ELon and hLon may be attributed to differences in the reversibility in ATP hydrolysis catalyzed by the two enzymes. Previously, it was demonstrated that ATP hydrolysis was irreversible in ELon (Thomas et al., 2010). In order to determine if ATP hydrolysis is reversible in hLon, we determined the number of isotopically-labeled oxygen atoms (^18^O) incorporated into the phosphate from an enriched reaction mixture by comparison to natural isotopic abundance. Rationale for the experimental design is summarized in Scheme 1. The Lon catalyzed ATPase reaction is conducted in the presence of ^18^O enriched aqueous buffer such that ^18^O will be incorporated into the inorganic phosphate generated from ATP hydrolysis. If ATP hydrolysis is irreversible, only one ^18^O enriched Pi (M+2) will be detected. If the ^18^O enriched Pi reforms ATP at the enzyme binding site and then become hydrolyzed by additional ${\text{H}}_{2}^{18}$O, then the molecular weight of inorganic Pi will be higher than M+2 as illustrated in Scheme 1. Therefore, the reversibility of ATP hydrolysis catalyzed by Lon could be deduced by determining the extent of ^18^O incorporated into the Pi product under steady-state enzyme catalysis condition. To facilitate the quantitative analysis of ^18^O incorporation into the Pi product, inorganic phosphate is derivatized by TMSDEA to yield a compound with a boiling point of 228–229°C that can be analyzed by GC/MS.

In this experiment, a control in which the natural ^18^O abundance of H_3_PO4 was determined. Table 3 shows the GC/MS approach accurately detected the expected natural abundance of ^18^O in H_3_PO4, thereby validating this detection method. Like bacterial Lon, human Lon possesses intrinsic ATPase activity that is stimulated by protein and certain peptide substrates. To evaluate the effect of protein substrate on the reversibility of the ATPase activity of human Lon, the ATPase reactions were conducted in the absence and presence of the lambda N protein (λN), which is degraded by human Lon and stimulates the ATPase activity (Maurizi, 1987). The results of ^18^O incorporation into inorganic Pi generated by hLon catalyzed ATP hydrolysis in the absence and presence of λN are shown in Table 3. Since the isotopic distribution of the molecular weight of trimethylsilylphosphate was enriched by M+2, one ^18^O was incorporated into the inorganic phosphate (Pi) generated from the hydrolysis of ATP. The ^18^O distribution in Pi product is consistent with the incorporation of one ^18^O, as no additional ^18^O incorporated Pi beyond the natural abundance, were detected. As shown in Table 4, in the absence of λN, an enrichment of 3 ± 1% in M+2 was detected (Table 4A, averaged of two trials shown in calculated enrichment). In the presence of the λN protein substrate, enrichment in M+2 of 19 ± 1% over the expected natural abundance was detected (Table 4B, averaged of the two trials shown in calculated enrichment). No significant enrichment was detected in the M+4 of Pi, which excludes the reformation of ATP by ^18^O labeled Pi generated during the first round of ATP hydrolysis. The detection of only one ^18^O incorporated into Pi product generated from hLon-catalyzed ATP hydrolysis in this study supports an irreversible ATPase mechanism. The observed difference in the calculated enrichment number (3 vs. 19%) between the stimulated vs. stimulated ATPase reaction is likely attributed to the relatively lower rate of ATP hydrolysis is in the intrinsic ATPase reaction. Such difference was also observed in the *E. coli* Lon catalyzed peptide-stimulated vs. intrinsic ATPase reactions.

**Table 3:** Calculated isotopic enrichment for control phosphate and for the potential incorporation of ^18^O into Pi from non-enzymatic hydrolysis of ATP.

	**Expected Abundance**	**Experimental Abundance**		
		**Control (H_3_PO_4_)**	**No enzyme Trial 1**	**No enzyme Trial 2**
M	100	100	100	100
M+1	24.63	24.30	24.29	24.25
M+2	12.65	13.04	12.68	12.80
M+3	2.12	2.17	2.15	2.30
M+4	0.5	0.65	0.56	0.58

**Table 4:** Experimental and calculated isotopic enrichment for the incorporation of 18O into Pi from hydrolysis of ATP by hLon in the presence of isotopically enriched H2O and in the absence **(A)** and presence **(B)** of λN.

	**MW of positive ion**	**Expected abundance**	**Experimental abundance**		**Calculated enrichment**	
			**Trial 1**	**Trial 2**	**Trial 1**	**Trial 2**
**A. INTRINSIC ATPase**						
M	299	100	100	100	0	0
M+1	300	24.62	24.31	24.65	0.32	0.04
M+2	301	12.65	16.24	15.14	3.59	2.49
M+3	302	2.12	3.39	3.32	0.39	0.59
M+4	303	0.5	1.04	1.09	0.091	0.28
						
**B**. λ**N-STIMULATED ATPase**						
M	299	100	100	100	0	0
M+1	300	24.62	25.09	25.80	0.46	1.17
M+2	301	12.65	32.78	31.60	20.13	18.95
M+3	302	2.12	6.36	6.55	0.72	0.24
M+4	303	0.5	2.56	2.10	0.48	0.80

### Effect of ADP on tryptic digest of Lon

**Figure 3:**
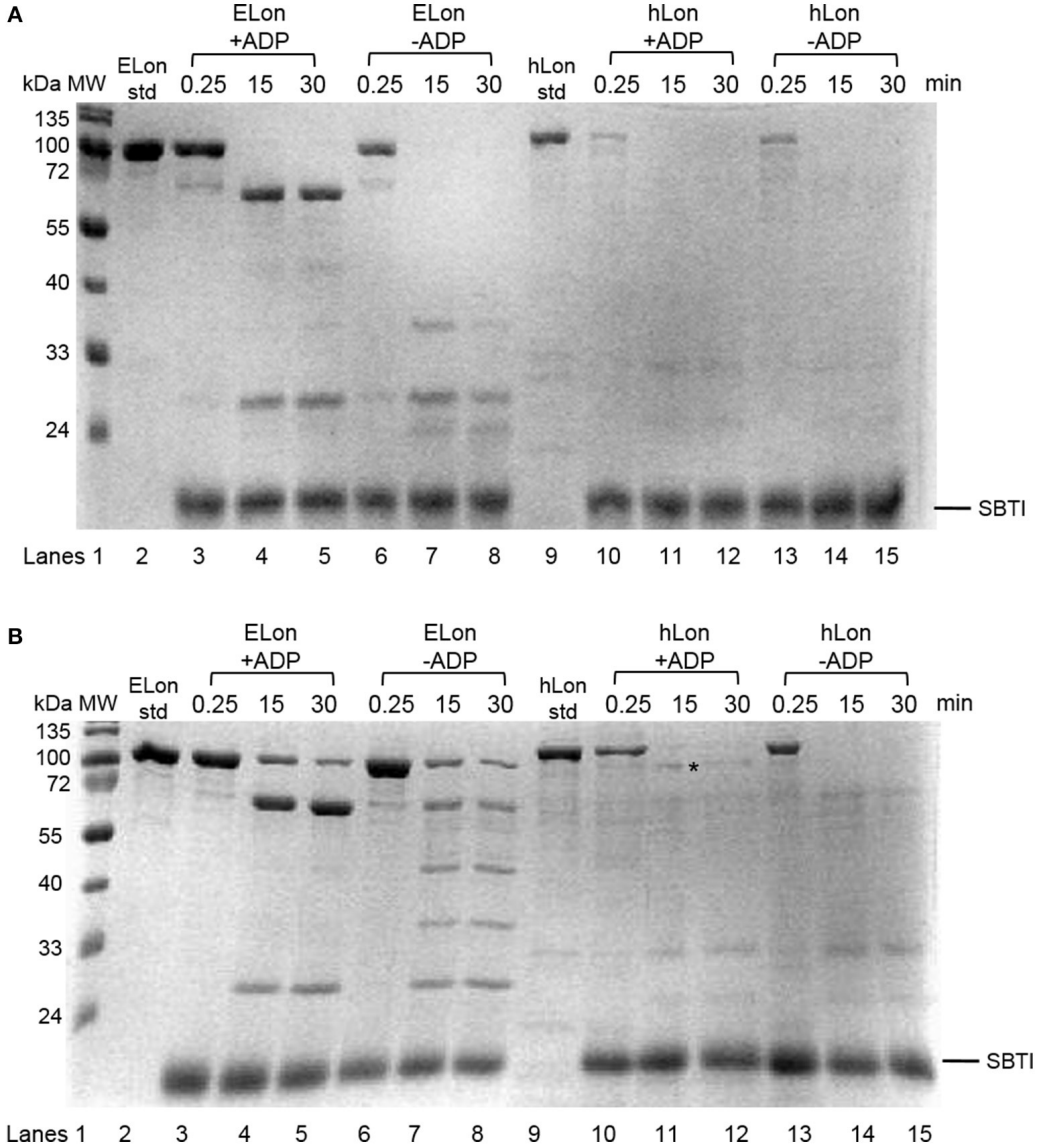
Limited tryptic digestion of bacterial and human Lon in the absence and presence of nucleotide. ELon and hLon were digested in the absence and presence of 1 mM ADP with limiting amounts of trypsin **(A)** 1/50 w/w and **(B)** 1/275 w/w and quenched at the indicated times with soybean trypsin inhibitor (SBTI) as described in Materials and Methods. The asterisk (^*^) between lanes 11 and 12 indicates the 72 kDa ADP-protected hLon fragment.

**Figure 4:**
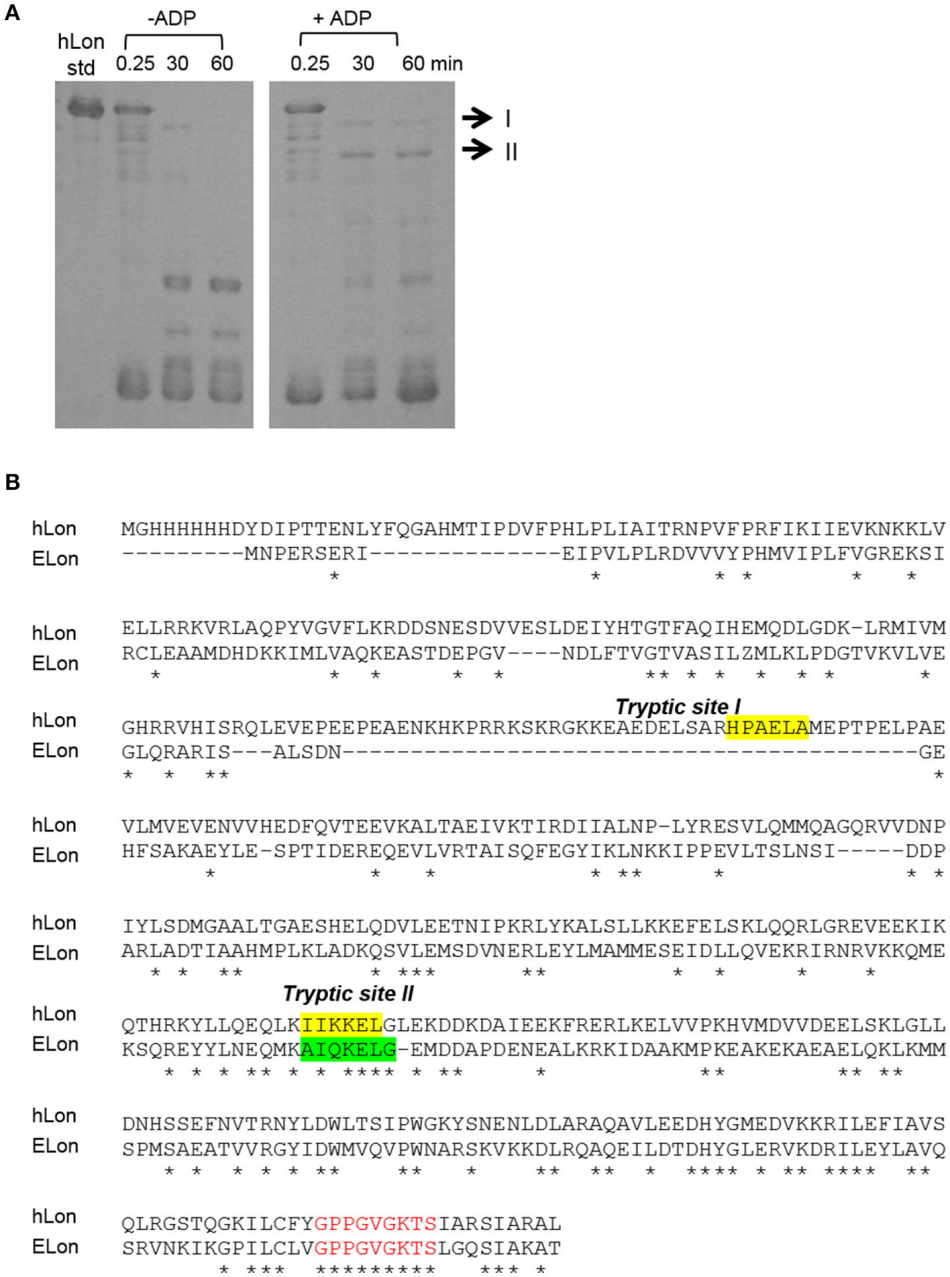
Identification of tryptic digest sites in hLon and ELon. **(A)** Three times the amount of trypsin-digested hLon in the absence and presence of 1 mM ADP compared to the results in Figure 3 was resolved by SDS-PAGE. The bands labeled I and II were subjected to Edman degradation. **(B)** Aligned sequences of hLon and ELon, with the tryptic digest sites determined by sequencing of bands I and II labeled. Conserved residues (^*^) and the conserved AAA+ protease Walker A motif (red) are indicated.

Previously, a limited tryptic digestion was examined to probe the functional role of nucleotide binding to Lon (Patterson et al., 2004). Upon binding to ADP, ELon became more resistant to tryptic digestion and yielded a 67 kDa Lon fragment consisting of the ATPase and protease domains but lacking the first 240 residues (26 kDa) of the amino terminal. Since our inhibition data showed that hLon bound to ADP with much lower affinity than ELon, we decided to utilize the same tryptic digestion assay to probe the interaction of hLon with ADP. Figure 3B shows the limited tryptic digestion profiles of hLon (1 μM) vs. ELon (1 μM) incubated in the absence and presence 1 mM ADP and digested by 1/50-fold (w/w) (Figure 3A) and 1/275-fold (w/w) (Figure 3B) trypsin under identical conditions (see Section Materials and Methods.) The first time point was obtained 0.25 min after initiating the reaction with trypsin before quenching an aliquot in SBTI and SDS loading buffer. The results indicated that hLon started to be degraded even before the aliquot was quenched but ELon was intact, both in the absence and presence of ADP. In the ELon profile, a 67 kDa fragment persisted at the 15 and 30 min time points only in the presence of ADP. By comparison, hLon was rapidly digested by 1/50-fold trypsin over Lon in the presence and absence of ADP and no specific ADP-protected fragment was detected in lanes 10–15 of Figure 3A, suggesting ADP does not protect hLon from tryptic digestion to produce two defined fragments as in the case of ELon. When the ratio of trypsin to Lon was reduced to 1/275 (Figure 3B), intact ELon in addition to the 67 kDa and 26 kDa ELon fragments were detected in the ADP treated reactions (lanes 4 and 5). In the absence of ADP, the intensity of the 67kDa ELon fragments was reduced and fragments corresponding to 42 and 37 kDa were detected. By comparison, very faint hLon fragments were detected in tryptic digestion time points of hLon treated with and without ADP. A very faint band corresponding to an apparent molecular weight of 72 kDa (labeled with ^*^ between lanes 11 and 12) was detected only in the time points containing ADP, suggesting this is an ADP-protected hLon fragment. To follow up on this observation, three times the amount of tryptic digested hLon sample treated with and without ADP were resolved with a 7.5% SDS-PAGE. As shown in Figure 4A; one hLon fragment, labeled II, was detected only in the ADP-treated reaction. The intensity of the fragment labeled I was stronger and persisted at the 60 min time point in the ADP treated reaction. Fragments I and II of hLon were sequenced by Edman degradation to identify the tryptic sites, which are summarized in Figure 4B. The tryptic digested site II matches up with the tryptic digestion site of ELon that was previously shown to be responsible for generating the 67 kDa ADP-protected ELon fragment shown in Figure 3A. The 72 kDa ADP-protected hLon fragment is consistent with the calculated molecular weight of the matured human mitochondrial containing the ATPase and proteolytic domain. Therefore, despite the longer hLon sequence and a 42% sequence homology, hLon and ELon bind to ADP and undergo at least one structural change that expose the same tryptic digestion site, suggesting the presence of at least one conserved structural change in the two enzyme homologs upon binding to ADP. However, the overall difference in the tryptic digestion patterns detected in the ELon vs. hLon shown in Figures 3A,B could be attributed to difference in structural dynamics, local conformational flexibilities and/or accessibility of tryptic sites in the respective proteins, and will require a higher resolution method for further clarification. An identical experiment utilizing 10 mM ADP in order to saturate hLon was also carried out to similar results (data not shown). The presence of a high amount of ADP, far more than would ever be present *in vivo*, did not protect hLon from digestion.

Certain bacteria, such as *Salmonella enterica* subspecies *enterica* serovar Typhimurium (*S. Typhimurium*), are responsible for causing a range of human diseases, such as gastroenteritis and typhoid fever. *Salmonella* Typhimurim Lon protease is required for systemic infection in mice, which is a common study model for *S*. Typhimurim infection in humans (Takaya et al., 2003). When Lon-deficient *S. Typhimurim* is administered as an oral vaccine in mice it has been shown to confer protection against subsequent infection by *S. Typhimurium* (Matsui et al., 2003). ELon and *S. Typhimurium* share >99% sequence identity (Johnson et al., 2008). In this study, we demonstrated that the binding of ADP for hLon and ELon differs significantly, suggesting that despite high sequence homology in the ATPase sites, there are mechanistic differences between the homologs. With the recent advances in high-throughput screenings of inhibitors as well as activity probes for kinases, the variations in ADP binding by bacterial vs. human Lon could be potentially exploited to develop selective inhibitors against the bacterial enzyme homologs.

## Summary

Lon has drawn significant biomedical interest since its discovery. In bacteria, Lon contributes to the pathogenicity of certain bacteria whereas in human, Lon contributes to the maintenance of mitochondria integrity. Therefore, the ability to identify unique features in bacterial Lon will benefit the development of antibiotic agents. In eukaryotes, Lon is located in mitochondria, where ATP is synthesized. Since the proteolytic activity of Lon is coupled with its ATPase activity, which yields ADP, knowing the effect of ADP on the proteolytic activity of eukaryotic Lon will help decipher the mechanism by which the activity of Lon is regulated in mitochondria. Driven by these goals, this study undertook a mechanistic approach, using comparable experiments performed on ELon, to evaluate the effect of ADP on the structure and function of the human homolog. Results generated from this study were directly compared with those obtained in ELon to identify difference between the two proteases. By monitoring the extent of ^18^O incorporation into the hydrolyzed inorganic phosphate product, we observed that hLon catalyzed ATP hydrolysis in an irreversible manner, which was the same in ELon. Despite showing comparable *k*_cat_ and *K*_m_ values in the ATPase activity, the *K*_i_ values of ADP toward the ATP-dependent peptidase activity of Elon were 300–1,500 times lower than those determined for hLon. Judging by the significant difference in protection from limited tryptic digestion in hLon incubated with ADP, we conclude that the mechanisms of ELon and hLon binding to ADP and/or ATP are different. In ELon, the binding interaction with ADP is strengthened by the removal of the gamma phosphate moiety whereas in hLon, such binding interaction is significantly weakened. Based on this observation, we propose that exploring the difference in the binding mechanisms of ADP in ELon vs. hLon will potentially serve as a viable strategy for developing selective inhibitors against Lon in pathogenic bacteria. Another significant finding of this work is the discovery that protein substrate-promoted ADP/ATP exchange mechanism existing in ELon is absent in hLon, as the *K*_i_ of ADP for hLon is >30-fold higher than the *K*_m_ of ATP. In mitochondria, the anticipated level of ATP is at least on millimolar level. Therefore, it is not likely that the proteolytic activity of hLon could be significantly affected by the concentration of ADP in the mitochondria. Given such consideration, the rate-limiting step governing the proteolytic activity of mitochondrial Lon as well as the mechanism that regulates its activity is unknown. Since specific mutations of human Lon have been shown to cause diseases such as CODAS (Strauss et al., 2015), we propose that a more thorough mechanistic study of wild-type vs. mutant hLon will be needed to advance our understanding on the role played by hLon in mitochondrial biology.

## Author contributions

IL designed the project, directed all experiments, analyzed and interpreted data, and wrote the manuscript. JF designed inhibition experiments, purified enzymes, synthesized peptides, analyzed and interpreted data, and wrote the manuscript. ZS performed limited tryptic digestion experiments. IC performed the ADP inhibition experiments, and limited tryptic digestion experiments, ^18^O exchange experiments and data analysis. YX designed and directed the ^18^O exchange experiment. SC performed the ^18^O mass spec data acquisition.

### Conflict of interest statement

The authors declare that the research was conducted in the absence of any commercial or financial relationships that could be construed as a potential conflict of interest.

## Abbreviations

Abz: aminobenzoic acid
ADP: adenosine 5′-diphosphate
ATP: adenosine 5′-triphosphate
DLU: density light units
DTT: dithiothreitol
Fmoc: 9-fluorenylmethoxycarbonyl
IPTG: Isopropyl β-D-1-thiogalactopyranoside
Mg(OAc)_2_: magnesium acetate
Ni-NTA: nickel nitrilotriacetic acid
ROS: reactive oxygen species
SDS-PAGE: sodium dodecyl sulfate-polyacrylamide gel electrophoresis
THF: tetrahydrofuran
Tris: tris(hydroxymethyl)aminomethane
TMSDEA: trimethylsilyl diethylamine
GC/MS: gas chromatography/mass spectrometry.
